# Evaluation of Arclight Frugal Smartphone Video Otoscopy

**DOI:** 10.1002/lary.32106

**Published:** 2025-03-06

**Authors:** Christoph R. Buhr, Katharina Bahr‐Hamm, Christopher Seifen, Johannes Pordzik, Jonas Eckrich, Thomas Murray, Christoph Matthias, Andrew Blaikie

**Affiliations:** ^1^ Department of Otorhinolaryngology University Medical Center of the Johannes Gutenberg‐University Mainz Mainz Germany; ^2^ School of Medicine University of St Andrews St Andrews UK

**Keywords:** ear canal, low‐resource, medical education, telemedicine

## Abstract

**Background:**

Frugal innovations are essential in low‐resource settings to provide cost‐effective medical solutions without compromising functionality. The Arclight is a robust, solar‐powered device that functions as both an ophthalmoscope and an otoscope, priced at approximately 12€ in low‐ and middle‐income countries (LMICs).

**Objectives:**

This study evaluates the Arclight's potential as a video otoscope when attached to smartphones, aiming to enhance telemedicine capabilities in ear examinations.

**Methods:**

Twenty otorhinolaryngology (ORL) specialists rated the standalone Arclight as well as its attachment to an iPhone 12 and a Samsung Galaxy A14 for ear examinations at the University Medical Centre in Mainz, Germany.

**Results:**

The standalone Arclight received the highest ratings overall, followed by the Arclight attached to a Samsung Galaxy A14. Attached to the Samsung Galaxy A14, the Arclight demonstrated significantly higher performance regarding ease of focus (*p* < 0.01), quality of view (*p* < 0.05), and zoom capabilities (*p* < 0.05) compared to the iPhone12 attachment. The iPhone12 setup performed lowest in all categories.

**Conclusion:**

The Arclight is an effective, low‐cost tool for ear examinations in low‐resource settings. While smartphone integration enhances its potential for telemedicine, performance varies depending on the smartphone used. The Samsung Galaxy A14 proved to be a more reliable option for tele‐otoscopy than the iPhone 12. Further optimization is needed to improve smartphone integration and address identified limitations, enhancing the Arclight's utility in telemedicine applications.

**Level of Evidence:**

NA.

## Graphical abstract

Frugal innovations are essential in low‐resource settings to provide cost‐effective medical solutions without compromising functionality. The Arclight, which functions as both an ophthalmoscope and an otoscope, is an effective, low‐cost tool for (video) ear examinations in low‐resource settings. While smartphone integration enhances its potential for telemedicine (video function), performance varies depending on the smartphone used.

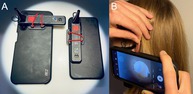

## 1 Background

Frugal innovations are essential in low‐resource settings to provide cost‐effective medical solutions without sacrificing functionality or performance. As defined by Bhatti et al. frugal innovations aim to “do more with less for the many”, addressing the needs of populations where traditional medical devices are often unaffordable or inaccessible [1]. Weyrauch et al. have emphasized the importance of balancing cost reduction with maintaining effectiveness in such innovations [2].

In the field of Otorhinolaryngology (ORL), accessible and affordable tools for ear examinations are particularly needed in low‐ and middle‐income countries (LMICs), where ear diseases and hearing impairment are prevalent but specialist care is scarce [3]. Early diagnosis and treatment are crucial to prevent hearing loss, underscoring the importance of developing effective diagnostic tools suitable for these settings.

Several approaches have been explored to facilitate ear examinations in low‐resource environments. Bhavana et al. investigated the use of standalone smartphone cameras for ear examinations, but these setups were limited by the need for wide ear canals with minimal curvature [4]. Cai et al. tested small, smartphone‐connected cameras inserted into the ear canal, but these often suffer from poor smartphone compatibility [5]. Additionally, Capobussi et al. examined three‐dimensionally printed otoscopes, but these devices were limited to ear examinations and lacked versatility [6].

**FIGURE 1:**
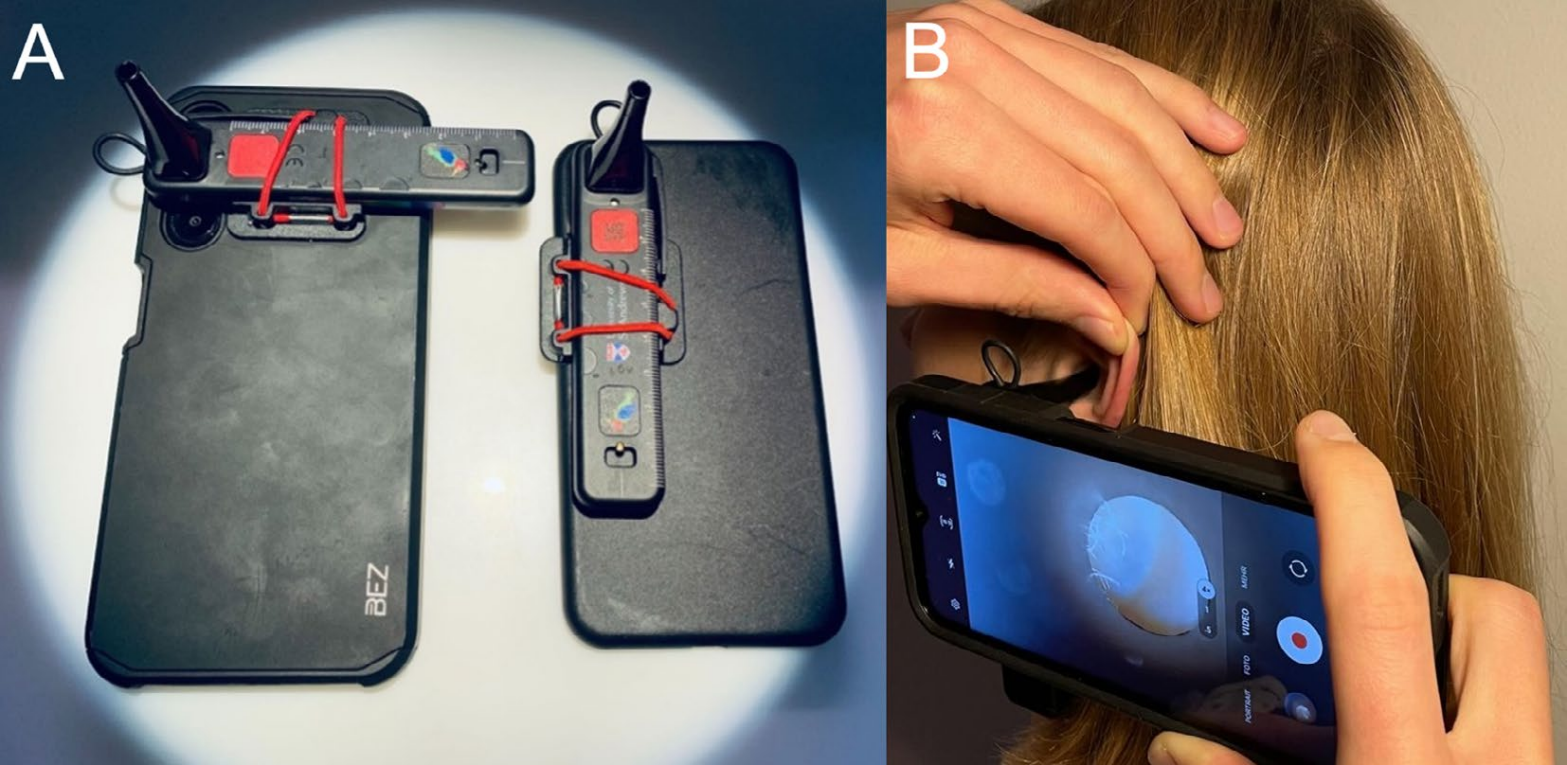
(A) The Arclight attached to a Samsung Galaxy A14 (left), attached to an iPhone 12 (right). (B) The Arclight attached to a Samsung Galaxy A14 during otoscopy.

In contrast, the Arclight device presents significant advantages. It is a robust, solar‐powered, and cost‐effective tool (approximately 12€ in LMICs) that functions as both an ophthalmoscope and an otoscope [7]. Its ability to operate without external power sources makes it particularly suitable for low‐resource settings. Furthermore, it can be attached to any smartphone via a sturdy adapter (see Figure 1), enhancing its utility for digital health applications, including telemedicine consultations. The widespread availability of smartphones in LMICs makes this a promising avenue for expanding access to ORL care through telemedicine.

This study is the first to explore the Arclight's potential as a video otoscope by evaluating its performance when attached to two different smartphones: an iPhone 12 and a Samsung Galaxy A14. Given the increasing penetration of smartphones in LMICs, [8] this study aims to determine whether such setups could provide a reliable solution for tele‐otoscopy, thereby enhancing access to ORL care through telemedicine.

## 2 Materials and Methods

The study involved 20 ORL specialists (8 consultants and 12 residents) from the Department of Otorhinolaryngology at the University Medical Center in Mainz, Germany. Each participant performed ear examinations on volunteers using three different configurations:

1. Standalone Arclight (Gold Standard): Using the Arclight standalone without a smartphone attachment.
2. Arclight with iPhone 12 Attachment: The Arclight is attached to an iPhone 12 using a custom adapter.
3. Arclight with Samsung Galaxy A14 Attachment: The Arclight is attached to a Samsung Galaxy A14 using the same adapter.

**FIGURE 2:**
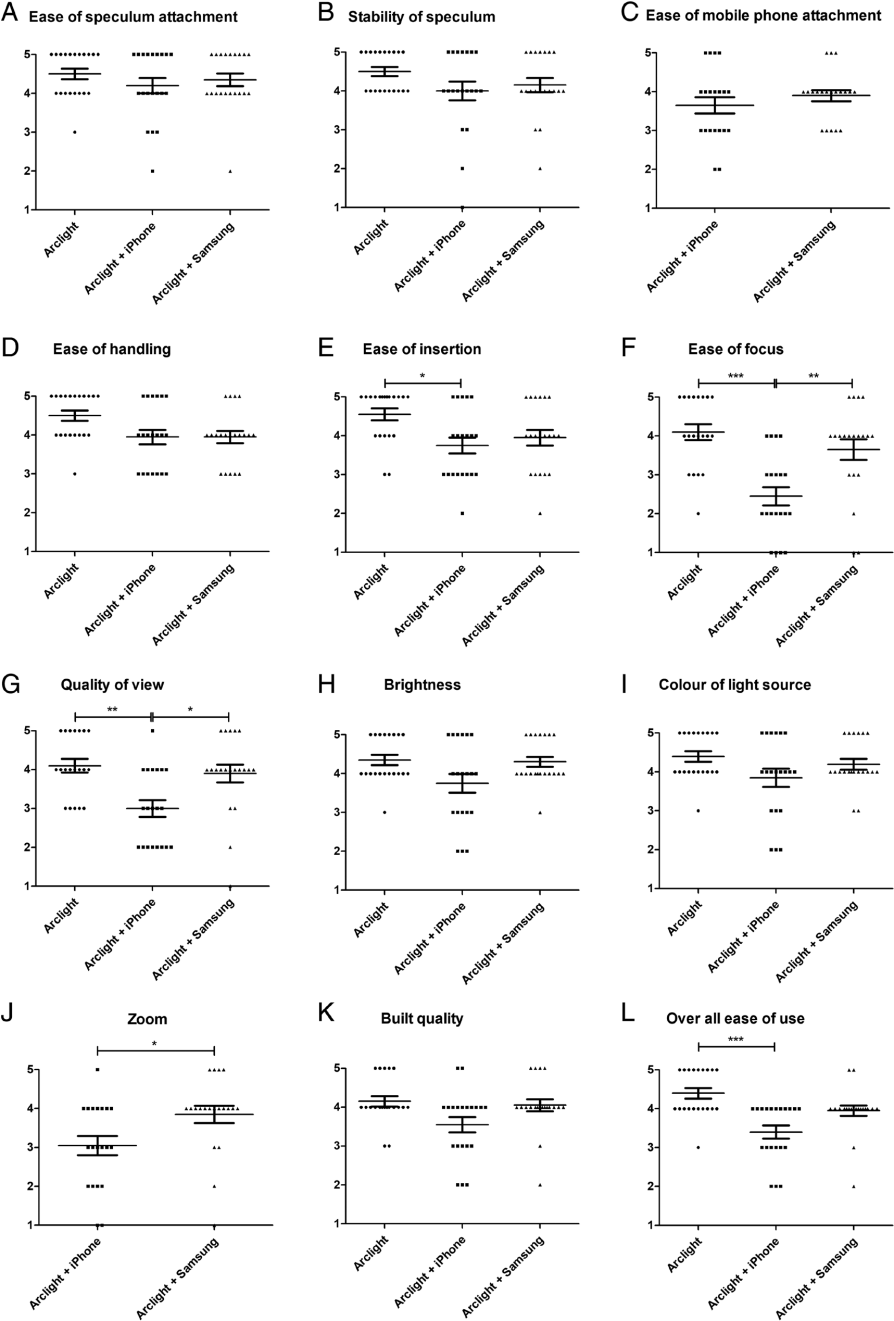
Ratings of ORL specialists on a 5‐point Likert scale (1 = very poor; 5 = excellent). Three group comparisons were performed using the Kruskal‐Wallis test, and two group comparisons were realized using the Mann‐Whitney test. *p < 0.05, **p < 0.01, ***p < 0.001.

After each examination, the specialists completed a detailed questionnaire assessing the performance of each configuration (10 key parameters for the standalone Arclight and 12 key parameters for each smartphone attachment), scored using a five‐point Likert scale (1 = very poor; 5 = excellent). An exemplary questionnaire is provided within the Supporting Information (Questionnaire Frugal Smartphone Video Otoscopy). The parameters evaluated are shown in Figure 2. Participants also provided free‐text responses to offer qualitative insights into the advantages and limitations of each setup. Before formal assessments, each specialist was given time to familiarize themselves with all three configurations. For each configuration, the specialists performed the examination and then completed the questionnaire.

The project received approval from the Ethics Committee of the Rhineland‐Palatinate Medical Association (DRKS‐ID DRKS00033209; 08.12.2023). Specialists evaluated each setup using a five‐point Likert scale, and they were also given an opportunity to provide free‐text comments on the advantages and disadvantages of each configuration. The data were analyzed using GraphPad Prism Version 5.01 for Windows, with normal distribution tested using the KS normality test. The Kruskal‐Wallis test and Dunn's multiple comparison test were used to compare the three groups, while the Mann–Whitney test was applied for two‐group comparisons.

## 3 Results

The standalone Arclight outperformed both smartphone‐attached setups across all evaluated parameters (see Figure 2). Ratings for the standalone Arclight were significantly higher than those for the iPhone 12 attachment in several areas:

– Ease of insertion (*p* < 0.05)
– Ease of focus (*p* < 0.001)
– Quality of view (*p* < 0.01)
– Overall ease of use (p < 0.001)

Specialists consistently praised the standalone Arclight for its ease of handling, compact size, and intuitive design, which contributed to its superior performance.

When comparing the smartphone setups, the Arclight attached to the Samsung Galaxy A14 received significantly higher ratings when attached to the iPhone 12 in key areas:

– Ease of focus (*p* < 0.01)
– Quality of view (*p* < 0.05)
– Zoom capabilities (*p* < 0.05)

Participants noted that the Samsung Galaxy A14 provided better focus stability, sharper image quality, and more effective zoom capabilities. This improved the diagnostic process and user experience. In contrast, the iPhone 12 attachment was criticized for difficulties with focus stability and poorer image quality.

In the free‐text comments, several specialists highlighted the standalone Arclight's ease of handling and insertion, appreciating its intuitive design and compactness. However, some limitations were noted:

– Speculum Sizes: The Arclight is provided with only one adult and one pediatric ear speculum, which may not accommodate patients with diverse ear canal sizes.
– Speculum Reuse: The specula are reusable and require thorough cleaning between patients. While this reduces plastic waste and is environmentally beneficial, it poses challenges for infection control, necessitating proper sterilization to prevent cross‐contamination.
– Auto Shut‐Off Feature: The device's 90‐s auto shut‐off feature sometimes interrupts examinations. Re‐activating the light requires unclipping the device from the smartphone, disrupting workflow and causing frustration.

**FIGURE 3:**
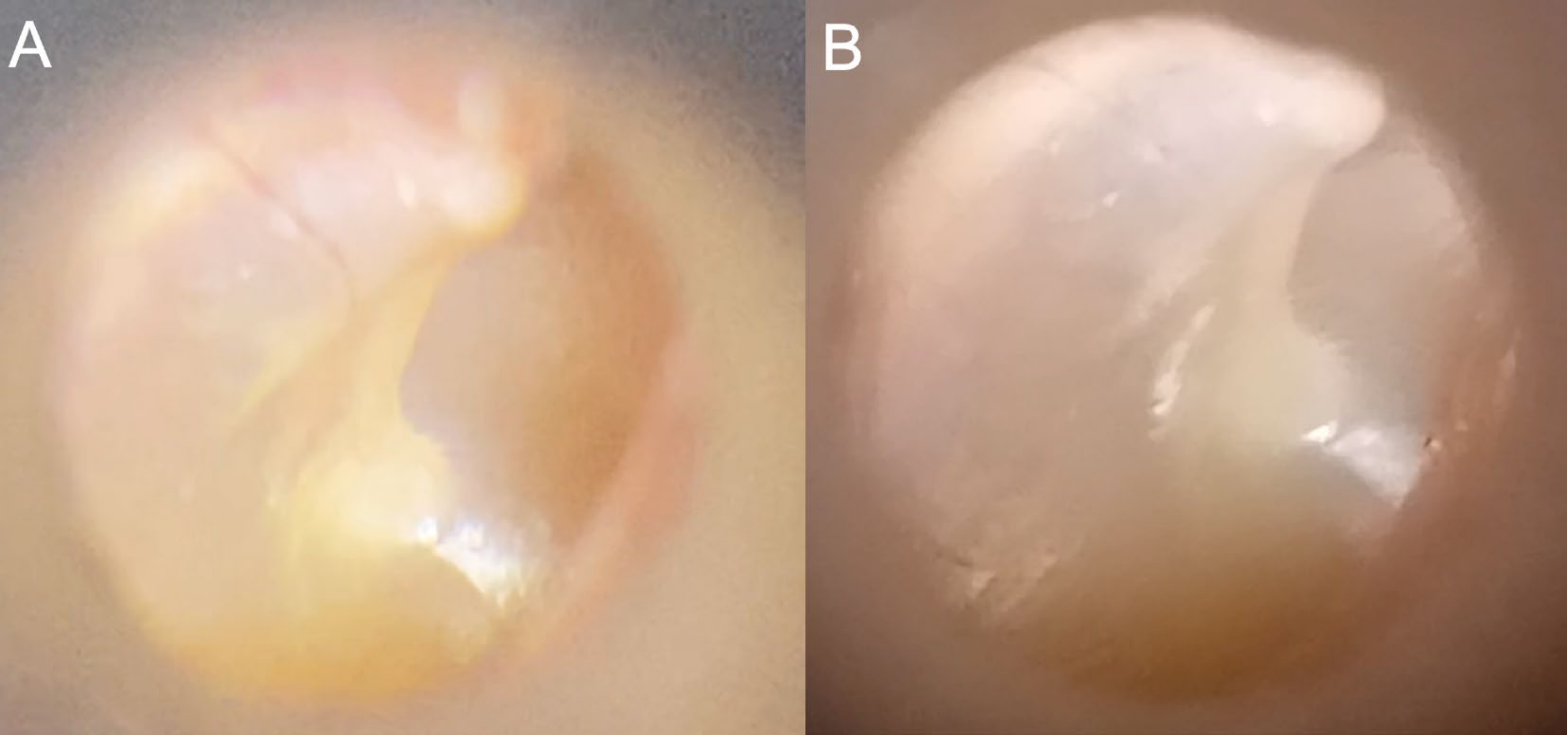
Exemplary screenshots of videos captured with the Arclight and attached to an iPhone 12 (A) and attached to a Samsung Galaxy A14 (B) respectively.

Screenshots of videos captured using the Arclight attached to both smartphones are shown in Figure 3, illustrating the differences in image quality and focus stability between the two setups.

## 4 Discussion

Previous studies have already shown that the Arclight performs comparably to traditional otoscopes [7, 9]. This study extends those findings by examining the device's performance when attached to smartphones for video otoscopy. The results of this study are significant for clinical practice in both high‐resource and low‐resource settings, highlighting the versatility of the Arclight in various contexts.

Our findings indicate that while the Arclight is an effective tool for ear examinations, integrating it with smartphones for telemedicine purposes presents challenges that vary depending on the smartphone used. The standalone Arclight provides the best overall user experience, suggesting that it is highly suitable for direct examinations in low‐resource settings.

The Arclight attached to the Samsung Galaxy A14 performed better than when attached to the iPhone 12, receiving higher ratings in critical areas such as ease of focus, image quality, and zoom capabilities. Participants attributed this to the Samsung Galaxy A14's better focus stability and camera compatibility with the Arclight. Better ratings of the Samsung Galaxy A14 for ease of insertion and overall ease of use might be attributed to the shape of the smartphone. Some participants preferred the angular edges of the Samsung Galaxy A14 over the rounded edges of the iPhone 12. Here, the perceived better overall ease of use might also explain the better rating of the quality of view. The better performance of the Samsung Galaxy A14 is particularly relevant for LMICs, where Android‐based smartphones like the Samsung Galaxy A14 are more affordable and widely available than more expensive devices like the iPhone 12.

Despite these advantages, limitations were noted with the smartphone setups. The increased bulkiness when attached to a phone affected handling and ergonomics. Additionally, issues with light stability and the device's auto shut‐off feature interrupted examinations, indicating areas for further optimization.

The limited availability of speculum sizes (only one size for adults and one size for children) and the need for thorough cleaning between uses were also highlighted as challenges. While the reuse of specula is environmentally friendly, it necessitates strict adherence to infection control protocols, which may be difficult in some low‐resource settings.

There are some limitations to this study. First, the study was conducted in a high‐resource setting with ORL specialists, which may limit the generalizability of findings to low‐resource environments where the Arclight is intended for broader use. This approach was chosen to ensure that evaluations were conducted by experienced professionals capable of providing nuanced feedback. Future studies involving general healthcare workers in low‐resource settings are planned to complement and validate these findings. Second, as this study is a user study, only subjective parameters were evaluated. However, subjective evaluations are valuable in this context as they reflect the real‐world experience of clinicians using the devices. Objective measures may not capture practical usability or clinical utility, which are critical for adoption in low‐resource settings. Third, the study design was not blinded, as participants were aware of the device or smartphone being used. Blinding was impractical because the physical characteristics and interface of each setup (e.g., smartphone model) were immediately evident to the users. Attempting to blind the users could have introduced logistical complexities and distracted from the primary aim of evaluating usability in realistic scenarios. Finally, the single ear speculum size (for adults) limits its versatility across different patient populations.

Despite a few limitations, this study underscores the potential of the Arclight as a tool for telemedicine in ORL care in LMICs but also highlights the need for further development to optimize smartphone integration. Enhancing compatibility with a wider range of smartphones, particularly those prevalent in LMICs, could improve its utility. Additionally, addressing the limitations identified could enhance the device's effectiveness for remote diagnostics. Moreover, newer generations of smartphones will provide better camera capabilities, further improving the overall performance. The Arclight's low cost (approximately 12€ in LMICs and 55€ in high‐resource settings) makes it a scalable solution for increasing access to ear examinations in resource‐limited environments [10]. The use of the Arclight could significantly enhance the diagnosis and management of conditions such as otitis media, which is prevalent in LMICs [3].

## 5 Conclusion

The Arclight is an effective, low‐cost tool for ear examinations, well‐suited to low‐resource settings due to its compact design, ease of use, and ability to function without external power sources. While integrating the Arclight with smartphones enhances its potential for telemedicine applications, performance varies depending on the smartphone used. The Samsung Galaxy A14 proved to be a more reliable option for tele‐otoscopy than the iPhone 12, likely due to better camera functionality.

To maximize the Arclight's utility in telemedicine, further work is needed to improve smartphone integration. This includes optimizing the device for use with a wider range of affordable smartphones common in LMICs, addressing issues related to handling, light stability, and user interface. Additionally, expanding the range of speculum sizes and developing strategies to ensure infection control without compromising environmental sustainability would enhance its applicability.

By addressing these challenges, the Arclight could play a significant role in improving access to ORL care in LMICs through telemedicine, ultimately contributing to the prevention of hearing impairment and the promotion of global health care.

## Ethics Statement

The project received approval from the Ethics Committee of the Rhineland‐Palatinate Medical Association (DRKS‐ID DRKS00033209; 08.12.2023).

## Conflicts of Interest

A.B. is employed part time by the University of St Andrews. The University owns a social enterprise subsidiary company which sells the Arclight diagnostic package to users in high‐resource countries. Profits are used to fund further development of tools as well as distribution and education exercises in low‐income countries.

## Supporting information


**Supporting Information.** Questionnaire frugal smartphone video otoscopy: (A) standalone arclight, (B) arclight + iPhone, (C) arclight + Samsung.
