# Supplementary material for: Evaluation of Arclight Frugal Smartphone Video Otoscopy
Source: Laryngoscope. 2025 Mar 6;135(8):2876–81. doi: 10.1002/lary.32106 (PMC12255373; doi:10.1002/lary.32106)
Supplement: Supplementary file 1 — Supporting Information. Questionnaire frugal smartphone video otoscopy: (A) standalone arclight, (B) arclight + iPhone, (C) arclight + Samsung. [file LARY-135-2876-s001.docx]

**Questionnaire Frugal Smartphone Video Otoscopy**

**A. Standalone Arclight**

1. Arclight


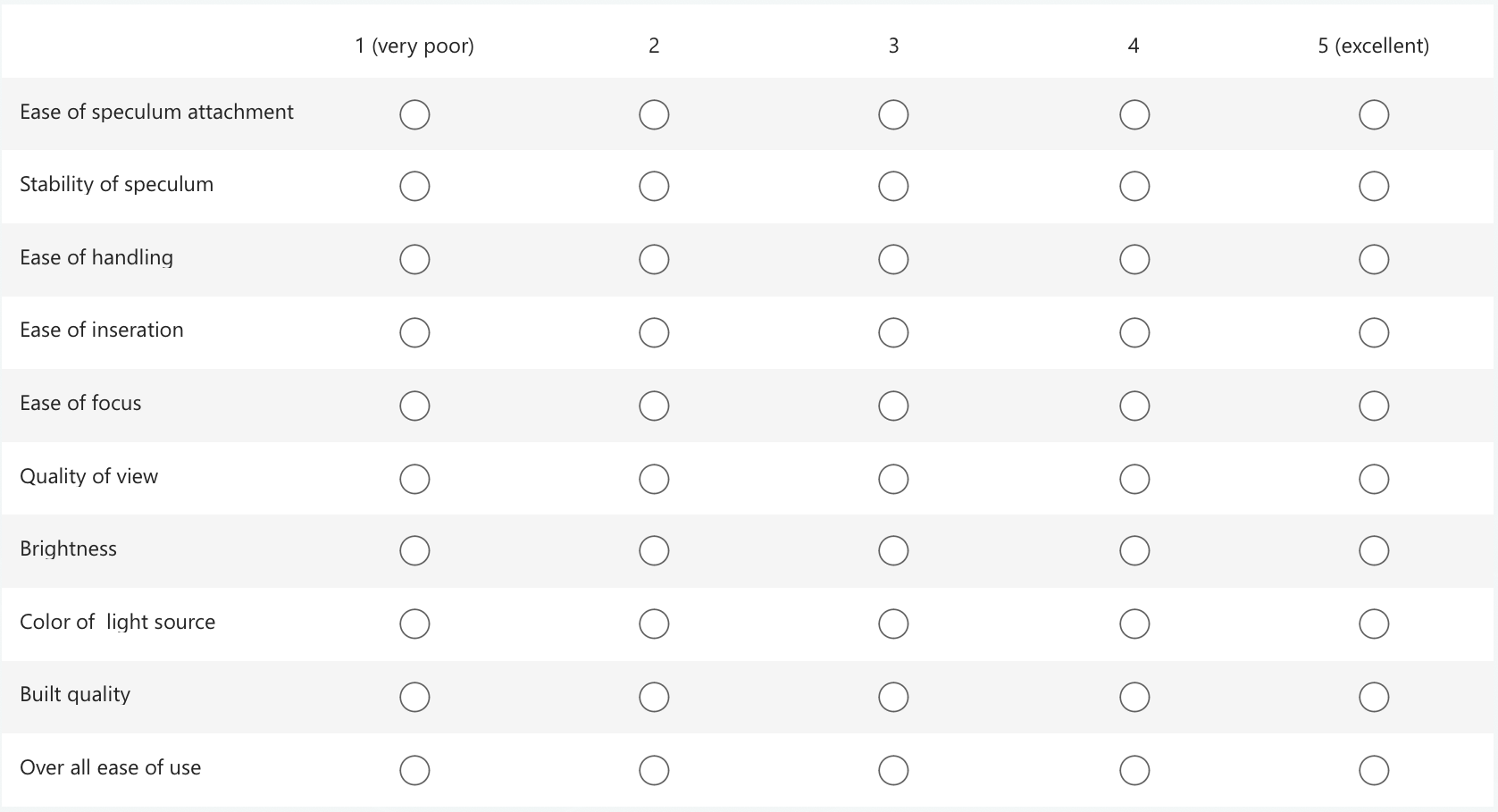


2. What did you find positive when using the Arclight?

3. What did you find negative when using the Arclight?

**B. Arclight + iPhone**

4. Arclight + iPhone


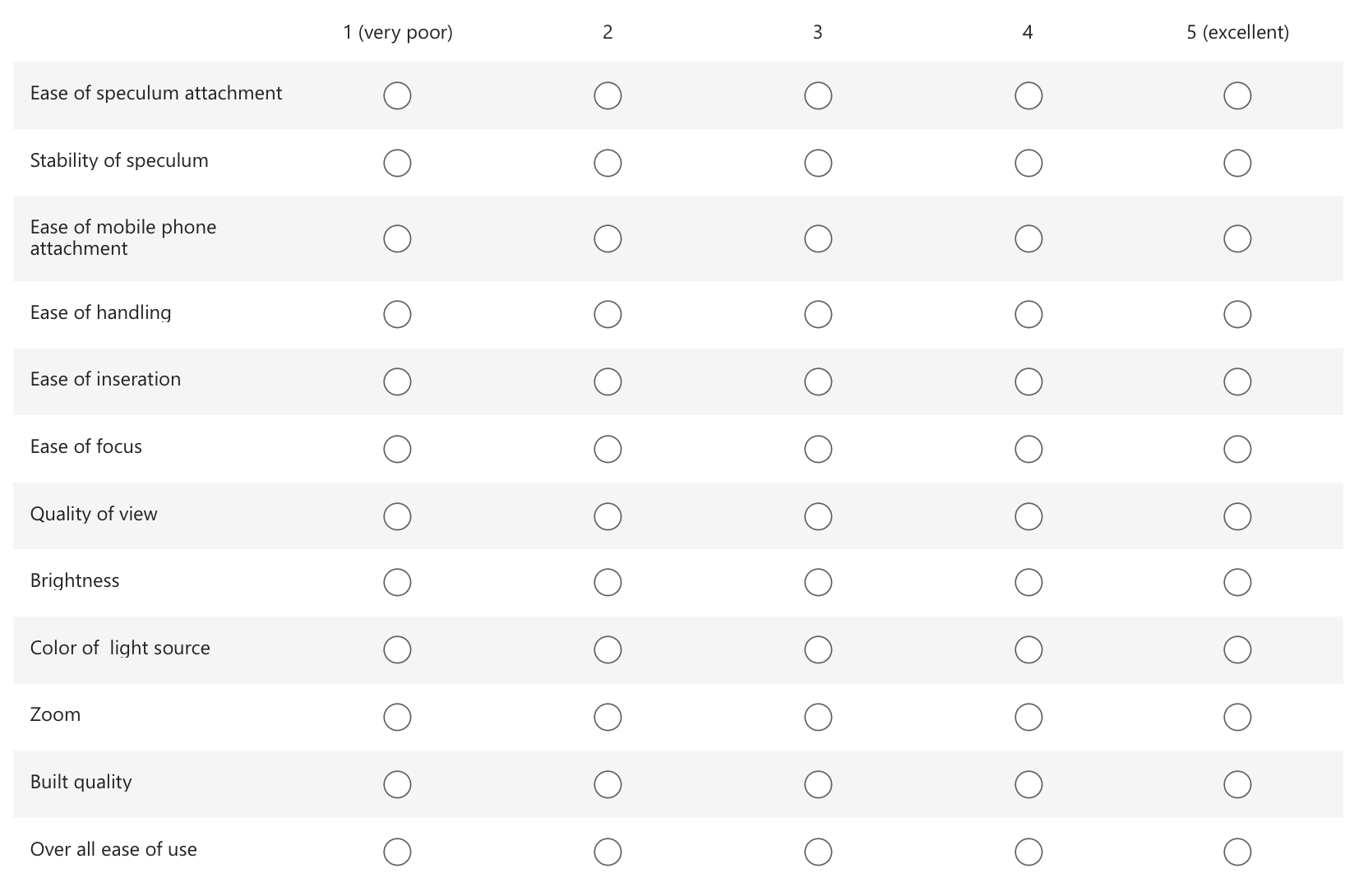


5. What did you find positive when using the Arclight + iPhone?

6. What did you find negative when using the Arclight + iPhone?

**C. Arclight + Samsung**

7. Arclight + Samsung


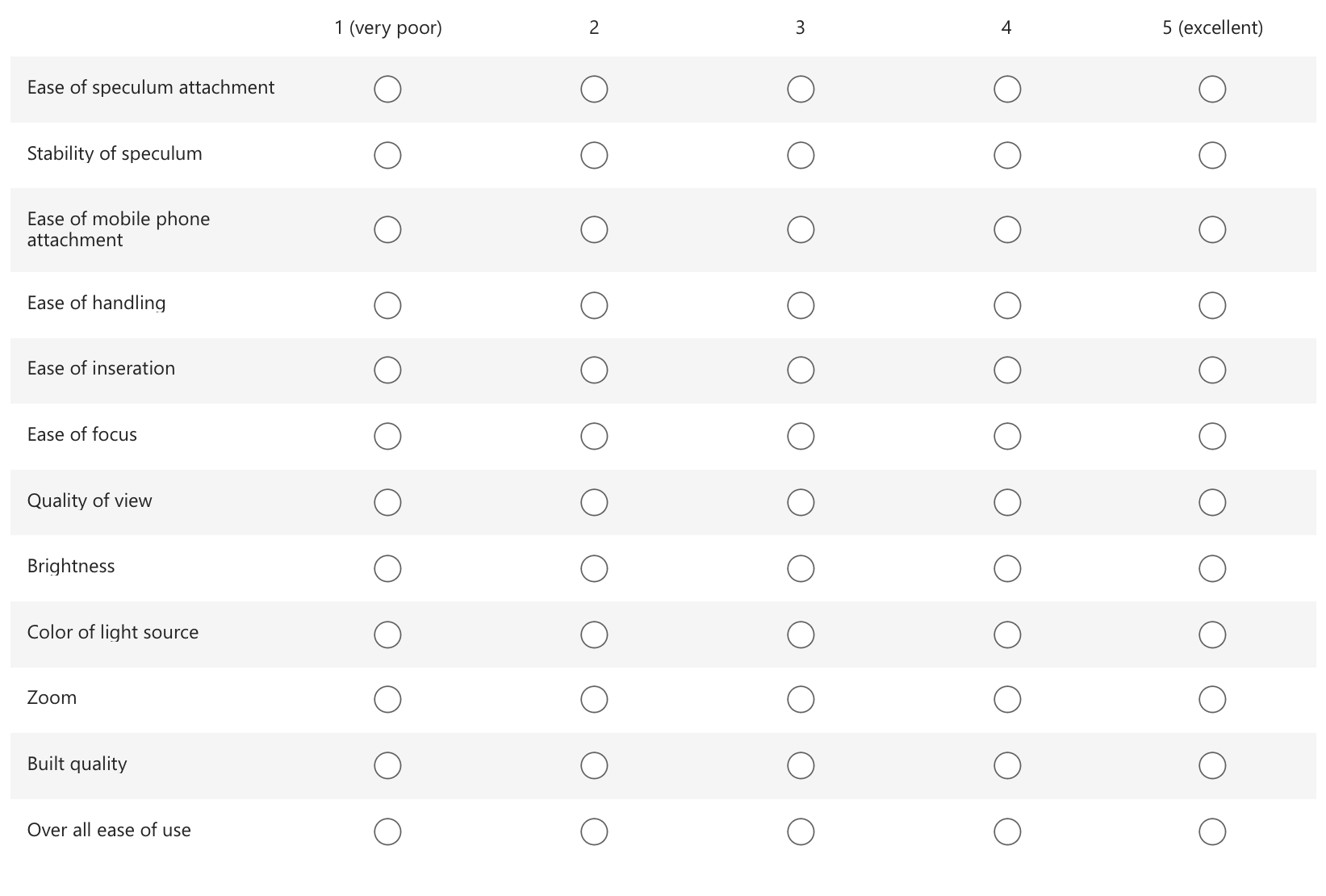


8. What did you find positive when using the Arclight + Samsung?

9. What did you find negative when using the Arclight + Samsung?
